# A Study for the Access to a Semi-synthetic Regioisomer of Natural Fucosylated Chondroitin Sulfate with Fucosyl Branches on *N*-acetyl-Galactosamine Units

**DOI:** 10.3390/md17120655

**Published:** 2019-11-21

**Authors:** Giulia Vessella, Serena Traboni, Anna V. A. Pirozzi, Antonio Laezza, Alfonso Iadonisi, Chiara Schiraldi, Emiliano Bedini

**Affiliations:** 1 Department of Chemical Sciences, University of Naples Federico II, Complesso Universitario Monte S. Angelo, via Cintia 4, I-80126 Napoli, Italy; giulia.vessella@unina.it (G.V.); serena.traboni@unina.it (S.T.); iadonisi@unina.it (A.I.); 2 Department of Experimental Medicine, Section of Biotechnology, University of Campania “Luigi Vanvitelli”, via de Crecchio 7, I-80138 Napoli, Italy; anna.pirozzi@unicampania.it (A.V.A.P.); chiara.schiraldi@unicampania.it (C.S.); 3 Department of Sciences, University of Basilicata, viale dell’Ateneo Lucano 10, I-85100 Potenza, Italy; antonio.laezza@unibas.it

**Keywords:** carbohydrates, polysaccharides, semi-synthesis, sulfation, glycosylation, fucose, fucosylated chondroitin sulfate

## Abstract

Fucosylated chondroitin sulfate (fCS) is a glycosaminoglycan found up to now exclusively in the body wall of sea cucumbers. It shows several interesting activities, with the anticoagulant and antithrombotic as the most attractive ones. Its different mechanism of action on the blood coagulation cascade with respect to heparin and the retention of its activity by oral administration make fCS a very promising anticoagulant drug candidate for heparin replacement. Nonetheless, its typically heterogeneous structure, the detection of some adverse effects and the preference for new drugs not sourced from animal tissues, explain how mandatory is to open an access to safer and less heterogeneous non-natural fCS species. Here we contribute to this aim by investigating a suitable chemical strategy to obtain a regioisomer of the natural fCS polysaccharide, with sulfated l-fucosyl branches placed at position *O*-6 of *N*-acetyl-d-galactosamine (GalNAc) units instead of *O*-3 of d-glucuronic acid (GlcA) ones, as in natural fCSs. This strategy is based on the structural modification of a microbial sourced chondroitin polysaccharide by regioselective insertion of fucosyl branches and sulfate groups on its polymeric structure. A preliminary *in vitro* evaluation of the anticoagulant activity of three of such semi-synthetic fCS analogues is also reported.

## 1. Introduction

Proteoglycans are one of the major, most ubiquitously distributed and most important components of the extracellular matrix and cell surfaces, playing several essential roles in a variety of biological events. They are very complex biomacromolecules, composed of a protein core on which are anchored from one to one hundred of polysaccharide chains, termed glycosaminoglycans (GAGs). These very often show a linear, highly negatively charged polysaccharide structure. Interestingly, some marine invertebrates present structurally unique GAGs [1], among which fucosylated chondroitin sulfate (fCS)—found up to now exclusively in the body wall of sea cucumbers (*Echinoidea*, *Holothuroidea*)—has been the most intensely studied in the last two decades. It attracted a constantly increasing interest for its activity in biological events related to inflammation, hyperglycemia, atherosclerosis, cellular growth, cancer metastasis, angiogenesis, and, above all, coagulation and thrombosis [2]. The anticoagulant and antithrombotic activity is observed also on antithrombin (AT) and heparin cofactor II (HC-II)-free plasmas, for which the most widespread and long-term used anticoagulant drug—unfractionated heparin—is inactive, due to some differences in the mechanism of action of fCS on the blood coagulation cascade [3,4]. Furthermore, oral administration of fCS retains its activity, because it is digested neither during its adsorption in the gastrointestinal tract nor by intestinal bacterial enzymes [5]. These features make fCS a very promising anticoagulant drug candidate for heparin replacement [6].

**Figure 1:**
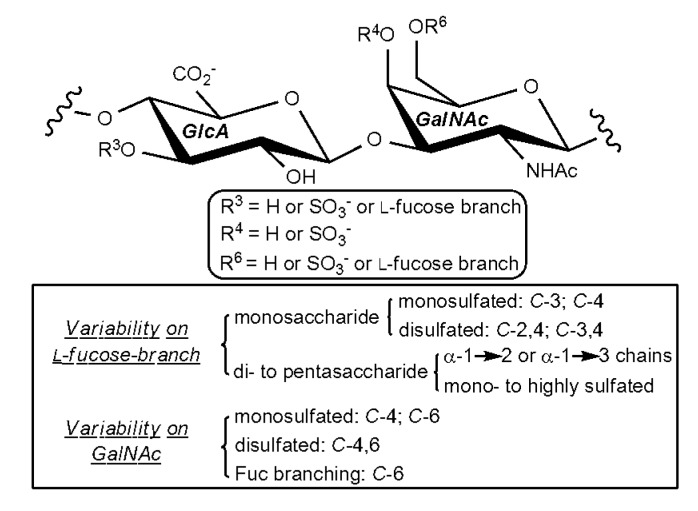
Structural variability of fCS from sea cucumbers.

From a structural point of view, fCS shares the same linear core backbone as chondroitin sulfate (CS) polysaccharide, with alternating *N*-acetyl-d-galactosamine (GalNAc) and d-glucuronic acid (GlcA) residues linked together through alternating β-1→3 and β-1→4 glycosidic bonds and sulfated to a different extent on their hydroxyls. The unique structural peculiarity of fCSs is the additional presence of variously sulfated fucose (Fuc) branches [7,8], which are essential for the observed biological activities [9,10,11]. Very often the branches are constituted of a single Fuc unit α-glycosidically linked at *O*-3 site of GlcA residues. Nonetheless, fCSs from some sea cucumbers species with slightly different structural features have also been found [12]. Indeed, *Ludwigothurea grisea* [13], *Eupentacta fraudatrix* [14], *Holothuria lentiginosa* [7], and *Holothuria mexicana* [15] show short Fuc oligosaccharides (from di- to pentasaccharides) instead of monosaccharide branches. For *Cucumaria frondosa* [16], *Actinopyga mauritiana* [17], *Holothuria scabra* [18] and, again, *Holothuria mexicana* [15], the presence of Fuc branches not only at GlcA *O*-3 sites but also at position *O*-6 of some GalNAc units has been proposed. Figure 1 summarizes the structural variability found up to now in natural fCSs.

It is worth noting that regardless of the structural details characterizing the fCS from different sea cucumber species, these polysaccharides very seldom display a homogeneous structure [7,19,20]. It is much more common to detect different repeating units within the same polymeric chains, thus resulting in heterogeneous structures that hamper detailed structure–activity relationship studies. Furthermore, together with the anticoagulant activity, native fCS polysaccharides exhibit some adverse effects, such as in promoting platelet aggregation, hypotension, and bleeding [21], that can be avoided by a significant decrease of the molecular weight (from 55–70 kDa to 8–12 kDa) [22]. To this aim, one approach is to obtain short, well-defined fCS structures by total synthesis [23,24,25] or by selective depolymerization of natural fCS polysaccharides [26]. As pure fCS oligosaccharide species, it was possible to obtain up to now only fragments not longer than a nonasaccharide [23], with an octasaccharide being demonstrated as the minimum structural unit responsible for anticoagulant activity [27]. In recent years, we have pursued an alternative strategy to synthesize fCS species, relying upon the chemical insertion of Fuc branches and sulfate groups at well-defined sites of a microbial-sourced chondroitin. This last polysaccharide shares the same linear backbone with CS but without any sulfate [28]. Such approach allowed the obtainment of a collection of semi-synthetic fCS polysaccharides differing for the sulfation pattern and the position of Fuc branches [29]. Some of these products exhibited a promising activity in some preliminary anticoagulant assays, such as AT-dependent activity against factor Xa and HC-II-mediated anti-factor IIa activity. Noteworthy, interesting results were obtained not only for some semi-synthetic polysaccharides with Fuc branches on GlcA sites, that closely resemble the most common structure from natural fCSs, but also for some regioisomeric species with Fuc units placed on GalNAc residues. Unfortunately, though the developed semi-synthetic strategies allowed the decoration of the polysaccharides with a very well-defined sulfation pattern, a limited regioselectivity was achieved in the Fuc branching which could only differentiate the GalNAc and GlcA units but not the single position of the same residue (GlcA *O*-2 *vs.* *O*-3; GalNAc *O*-4 *vs.* *O*-6). To overcome this limitation, we have decided to re-investigate the semi-synthetic strategies towards the obtainment of fCS polysaccharides with a precise regiocontrol not only concerning the sulfation pattern but also the Fuc branching position. In particular, a study of a semi-synthetic access to a fCS polysaccharide with Fuc branches exclusively placed at a single site of GalNAc units is here presented.

## 2. Results and Discussion

### 2.1. Semi-Synthesis of fCSs

**Scheme 1:**
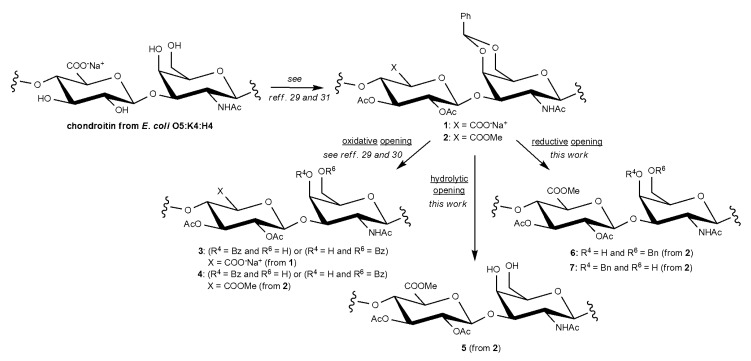
Protecting groups pathways potentially accessible on chondroitin via O-4,6 GalNAc benzylidene ring cleavage.

A differentiation between GalNAc and GlcA hydroxyls in *Escherichia coli* O5:K4:H4 sourced chondroitin could be easily done by protection of the diol on GalNAc units with a benzylidene ring [30], followed by acetylation of GlcA diol. This two-step sequence has been demonstrated to work very well on both chondroitin itself [31] and its derivative with GlcA carboxyl unit protected as methyl ester [29] to give polysaccharide derivatives **1** and **2**, respectively (Scheme 1). It is very well known in synthetic carbohydrate chemistry that benzylidene rings placed on 1,3-diols, such as on *O*-4,6 positions of *galacto*-configured sugars, can be removed employing different kinds of reactions. Both a hydrolytic and a hydrogenolytic cleavage restore the original diol. A reductive opening gives the protection of one of the two hydroxyls as benzyl ether and releases the alcohol at the complementary position in a regioselective way that depends on the employed reaction conditions. An oxidative cleavage affords the protection of one of the two hydroxyls as benzoyl ester and releases the alcohol at the complimentary position in a non-regioselective way, thus giving a mixture of both the possible isomers [32]. The last reaction has been already applied on chondroitin derivatives **1** and **2**. Together with the additional chemical steps, it has allowed for the first semi-synthetic method for accessing non-animal sourced CS-A,C [31] and non-regioselectively fucosylated fCS [29,33], respectively. In order to gain a higher Fuc branching regioselectivity in the semi-synthesis of fCS polysaccharides, alternative methods for benzylidene cleavage based on hydrolytic or reductive reactions were here investigated (Scheme 1).

**Scheme 2:**
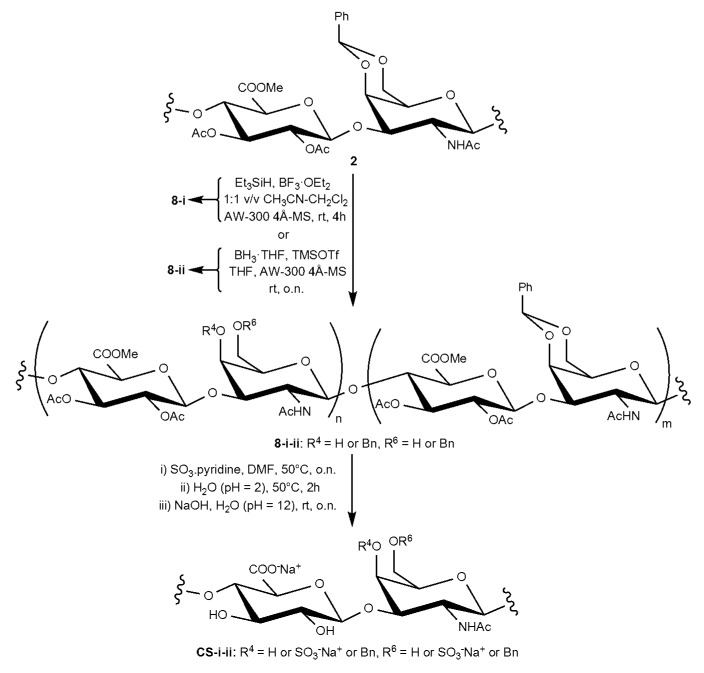
Semi-synthesis of **CS-i**–**ii** polysaccharides through benzylidene reduction approach.

The reductive opening methods already developed for the cleavage of benzylidene rings on monosaccharides or short oligosaccharides are based on borane- or silane-type reagents. Among the several variants that have been reported [32], we focused our attention on those showing the following features, necessary for an application on chondroitin polysaccharide derivative **2**: [29] (i) a high regioselectivity on *galacto*-configured sugars, and (ii) the possibility to avoid very low temperatures (e.g., −78 °C) for gaining a satisfying regioselectivity in benzylidene opening. The latter feature is desirable because it is highly unlikely that polysaccharide **2** is soluble in any solvent at very low temperatures. The methods employing either a borane-type reagent such as BH_3_·THF complex in THF under TMSOTf catalysis [34] or a silane-type reagent such as Et_3_SiH in CH_3_CN under BF_3_·OEt_2_ catalysis [35], were selected. They were tested on chondroitin polysaccharide **2** under strict anhydrous conditions ensured by an argon atmosphere and the presence of 3Å MS in the reaction mixture. Even if the selected methods are known to guarantee a high yielding benzylidene reductive opening to 4-benzylated derivatives on *galacto*-configured species [34,35], the resulting polysaccharides **8-i** and **8-ii** showed in their ^1^H NMR spectrum a residual signal related to the benzylidene methine proton at *δ*_H_ 5.45 ppm. Nonetheless degree and regioselectivity of modification could be not easily evidenced by NMR. In order to mark the positions of the liberated hydroxyls in **8-i**–**ii**, the polysaccharides were subjected to sulfation with SO_3_·pyridine complex in DMF, followed by deprotection under acid and then alkaline hydrolytic conditions. Such semi-synthetic sequence should have given, after purification by dialysis, partially *O*-benzylated chondroitin sulfate polysaccharides **CS-i**–**ii** (Scheme 2).

Instead, 2D-NMR spectroscopy investigation on these products revealed, by comparison with literature data [36,37], that signals of neither mono-sulfated disaccharide subunits (CS-A or CS-C having a single sulfate at position 4 or 6 of GalNAc units, respectively) nor benzyl ether moieties could be detected. Surprisingly, unsulfated (CS-0) and 4,6-*O*-disulfated subunits (CS-E) were only found, in 68:32 and 97:3 ratios for **CS-i**–**ii**, respectively, as measured by relative integration of GalNAc *O*-6 methylene signals for CS-0 and CS-E units (at *δ*_H/C_ 3.71/61.0 and 4.15–4.21/75.9 ppm, respectively) in DEPT-HSQC spectra (Figures S3 and S4). Unsulfated subunits could derive from the GalNAc residues of **8-i-ii** still carrying a benzylidene ring, according to their residual presence detected in the ^1^H NMR spectrum of **8-i**–**ii**. The presence of minor CS-E subunits could be explained with a hydrolytic—instead of reductive—mechanism operating during the benzylidene cleavage reaction. This is supposedly due to the presence of residual water molecules entrapped in the 3D-structure of polysaccharide **2** that cannot be eliminated even after co-evaporation of the polysaccharide with dry toluene, a prolonged vacuum-desiccation and finally the use of molecular sieves in the reaction vessel.

**Figure 2:**
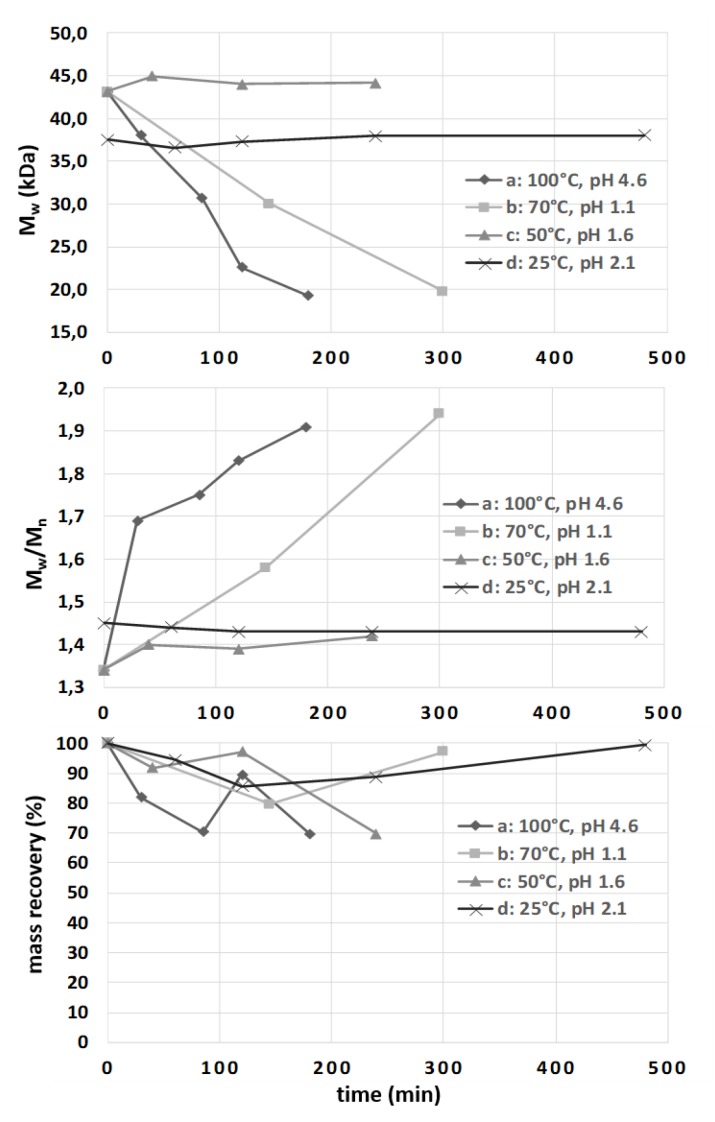
Stability data (up: molecular weight; middle: polydispersity; down: percentage mass recovery) of chondroitin polysaccharide chain to hydrolysis reactions: (a) acetic acid in water, 100 °C, pH 4.6; (b) aq. HCl, 70 °C, pH 1.1; (c) aq. HCl, 50 °C, pH 1.6; (d) self-hydrolysis of chondroitin in its acid form [30], 25 °C, pH 2.1.

Since the reductive opening of the benzylidene gave, instead, the reinstatement of the diol with a low degree of substitution, we tried to enhance the efficiency of this conversion. To this aim, some reactions commonly used in synthetic carbohydrate chemistry for the cleavage of benzylidene rings to restore the original diols, such as hydrogenolysis and hydrolysis, were tested. The former was not considered further because it typically employs a heavy metal, such as palladium, on an activated charcoal as catalyst, and is therefore expensive, toxic and hard to be completely removed from a polysaccharide product. Furthermore, this is a heterogeneous reaction that often gives low yield when—as for chondroitin derivative **2**—there is a high number of benzylidene rings to be removed and the saccharide substrate is complex and/or very long-chained such as a polysaccharide. On the contrary, hydrolytic cleavage of a benzylidene is much easier, greener and less expensive than hydrogenolysis, as it just requires aqueous acid solutions. Nonetheless, in the case of a polysaccharide, the reaction conditions must be finely tuned in order to achieve the cleavage of the benzylidene acetals with no significant breakage of the glycosidic bonds of the polysaccharide chain. To this aim, we preliminary studied the stability of the native chondroitin polysaccharide [28] chain to hydrolysis reactions performed under different temperature and pH conditions. Weight-averaged molecular mass (*M_w_*) and polydispersity—as ratio between *M_w_* and number-averaged molecular mass (*M_n_*)—were measured by high-performance size exclusion chromatography combined with a triple detector array (HP-SEC-TDA) [38,39,40], on aliquots taken from the reaction mixtures at different times and then dialyzed (3.5 kDa cut-off) at low temperature (4 °C) before HP-SEC-TDA measurements (Figure 2). The data clearly showed that the temperature played an important effect in shortening the polysaccharide chain and increasing the polydispersity, with a threshold of 50 °C not to be surpassed in order to guarantee the integrity of the polymer.

With this preliminary result in mind, we monitored the benzylidene acetal *vs.* glycosidic bond cleavage on chondroitin derivative **2** under hydrolytic conditions that could be adapted to such water insoluble, fully protected polysaccharide. Two different reactions were conceived. In the first one a concentrated aqueous solution of acetic acid was employed (90% *w*/*w* acetic acid in water, resulting in a solution with a protic activity approximately equal to that of reaction c in Figure 2). Under similar conditions, the selective cleavage of 4,6-*O*-benzylidene rings on complex *N*-glycan oligosaccharide derivatives [41] and then, during the development of this work, also on chondroitin oligosaccharide derivatives [42] have been reported. In the second reaction, **2** was treated with an organic Brønsted acid such as (+)-camphor-10-sulfonic acid (CSA) in acetonitrile (ACN) in the presence of an acetal exchange reagent (1,4-dithiothreitol, DTT). Such a very mild method for the selective cleavage of acetals and ketals, including benzylidene rings, from complex carbohydrate substrates has been published during the development of this work [43]. Aliquots were taken from both the reaction mixtures at different times, dialyzed (3.5 kDa cut-off) at 4 °C and, after freeze-drying, mass recovery and the degree of benzylidene cleavage (DBC) were evaluated. The latter was estimated from the integral areas measured for the residual benzylidene ring and methine proton signals at *δ*_H_ 7.5–7.2 and 5.45–5.35 ppm, respectively, and the *O*- and *N*-acetyl signals at *δ*_H_ 2.0–1.7 ppm in the ^1^H NMR spectrum, according to Equation (1) (see also Figures S5 and S6).

(1) $$DBC=\left(1-\frac{\frac{I\;\left(PhCH\right)}{\frac{I\;\left(Ac\right)}{9}}+\frac{\frac{I\;\left(PhCH\right)}{5}}{\frac{I\;\left(Ac\right)}{9}}}{2}\right)*100$$

**Figure 3:**
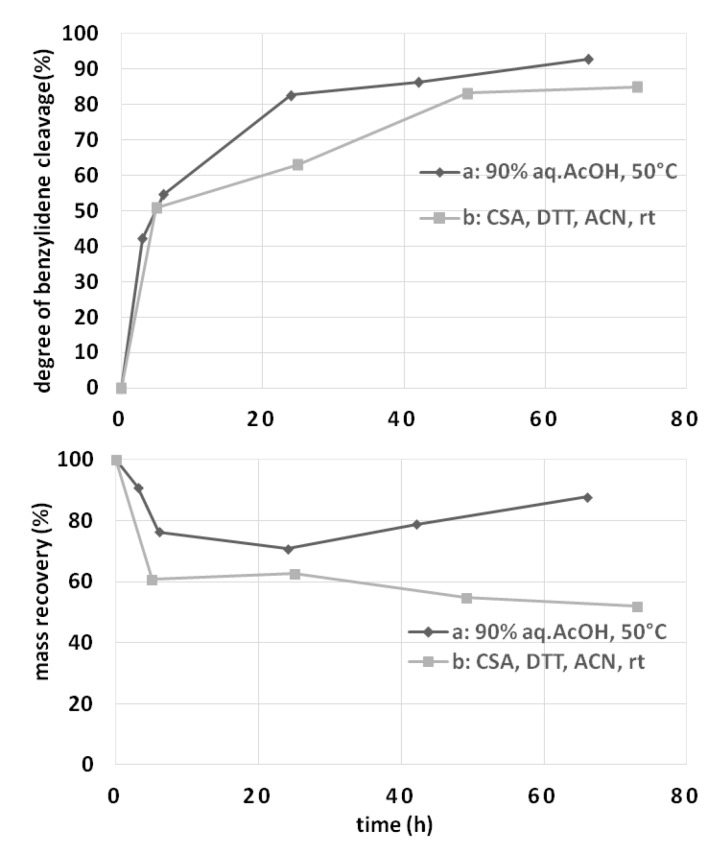
Conversion of polysaccharide **2** into **5** (up: percentage degree of cleaved benzylidene; down: percentage mass recovery) by: (a) 90% aq. acetic acid, 50 °C; (b) CSA, DTT, ACN, 25 °C.

**Scheme 3:**
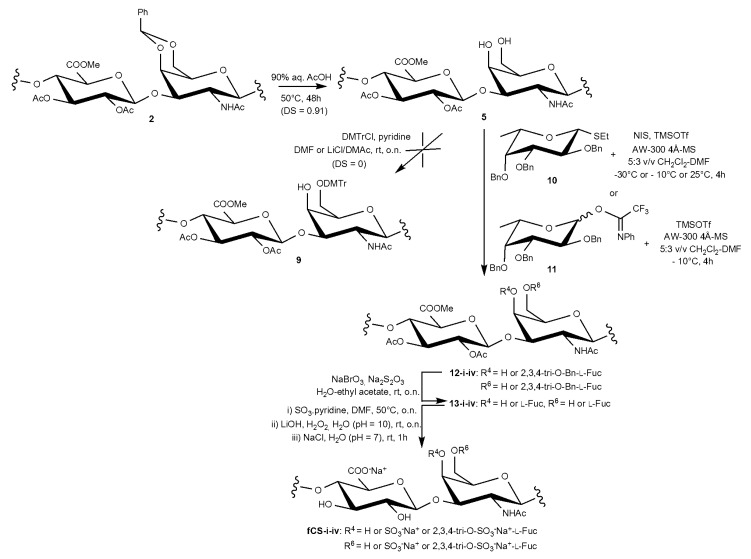
Semi-synthesis of **fCS-i**–**iv** polysaccharides fucosylated on GalNAc units through benzylidene hydrolysis approach (DS = degree of substitution).

The obtained data are summarized in Figure 3. The two reactions clearly afforded benzylidene cleavage with a very similar behavior, nonetheless the method employing aqueous acetic acid was preferred not only due to a slightly higher DBC and mass recovery but also for its cheapness. In particular, derivative **5** with a percent degree of restored diol equal to 92% was obtained by treating **2** with 90% aq. AcOH at 50 °C for 48 h. Polysaccharide **5** was then subjected to test reactions for the selective protection of the diol, in order to obtain a derivative with a single free alcohol per repeating unit that might be suitable for single site Fuc branching of GalNAc units (Scheme 3). In particular, the high steric demand and the ease of removal of 4,4′-dimethoxytrityl (DMTr) ethers justify their wide use as protecting groups for primary hydroxyls not only on simple carbohydrates but also on polysaccharides [44]. Unfortunately, the reaction of **5** with DMTr chloride (DMTrCl) in the presence of pyridine using DMF or LiCl in dimethylacetamide (DMAc) as solvent system [45] gave no derivatization at all. This was clearly indicated by the absence of aromatic signals in addition to the residual benzylidene ones in the ^1^H NMR spectrum of the polysaccharide recovered from the reaction mixture by precipitation.

As an alternative strategy to Fuc branching at a single site of GalNAc units, polysaccharide **5** was employed directly in glycosylation reactions with two fucosyl donors under different conditions. The hypothesis is that the primary hydroxyl of GalNAc 4,6-diol is much more nucleophilic than the secondary alcohol at position *C*-4. Furthermore, the latter has an axial orientation and a *cis*-glycosylation at the adjacent *C*-3 position. Both these features should be additional detrimental factors for its reactivity. Glycosylation of diol **5** was firstly tested with 2,3,4-tri-*O*-benzyl ethyl thiofucoside **10** [46] at different temperatures ranging from −30 to 25 °C using *N*-iodosuccinimide (NIS)/TMSOTf as thioglycoside activator system and a 5:3 *v/v* CH_2_Cl_2_-DMF as α-stereodirecting solvent mixture [33,47], that also allowed to conduct the reaction under homogeneous conditions. Since 2,3,4-tri-*O*-benzyl fucosyl *N*-phenyl-trifluoroacetimidate **11** [48] is also known to act as an efficient glycosyl donor in fucosylations [49], glycosylation of diol **5** was performed also with this Fuc donor. Polysaccharide products **12-i**–**iv** obtained by precipitation with suitable solvents from the crude glycosylation mixtures showed rather complex ^1^H NMR spectra (Figurs S7–S10) that impeded any structural characterization. Therefore, they were directly transformed into the target fCSs by a sequence of additional steps. Firstly, the Bn protecting groups on putative Fuc branches were oxidatively cleaved with NaBrO_3_ and Na_2_S_2_O_3_ in a H_2_O/ethyl acetate mixture [50] to give derivatives **13-i**–**iv** with three free hydroxyls on Fuc branches. The liberated alcohol moieties were then sulfated together with the residual non-fucosylated GalNAc sites under standard conditions with the SO_3_·pyridine complex in DMF at 50 °C [51]. Finally, alkaline hydrolysis of the ester protecting groups, followed by purification of the polysaccharides by dialysis and filtration on a C-18-silica reverse phase plug, furnished semi-synthetic **fCS-i**–**iv** in 50–58% weight yield (over five steps, calculated from **5**; see Table 1).

### 2.2. Structural and Preliminary Biological Characterization of Semi-synthetic fCSs

Integration of the ^1^H NMR spectra of semi-synthetic fCSs allowed the determination of some structural parameters such as degree of fucosylation (DF), 6-*O*/4-*O*-fucosylation ratio (glycosylation regioselectivity) and α-/β-linked Fuc ratio (glycosylation stereoselectivity). DF was evaluated as the ratio between the H-6 Fuc methyl (*δ* = 1.35–1.20 ppm) and the *N*-acetyl signal (*δ* = 2.10–1.95 ppm) integrations (Figure S11 and Table 1). By conducting the glycosylation between chondroitin acceptor **5** and thioglycoside donor **10** at −30 °C, DF of the final semi-synthetic **fCS-i** was 0.21. This value did not change (0.20) for **fCS-ii**, obtained by a glycosylation at −10 °C, and only a slight enhancement was observed for **fCS-iii** (0.29), afforded by increasing the reaction temperature at 25 °C. Instead, a very low DF (0.04) was detected for **fCS-iv**, derived from the glycosylation with *N*-phenyl trifluoroacetimidate donor **11** at −10 °C.

Fuc branching regioselectivity between GalNAc *O*-4 and *O*-6 positions was measured by integration of DEPT-HSQC spectra of **fCS-i**–**iii**, assuming that the signals to be compared displayed similar ^1^*J*_CH_ coupling constants and that a difference of around 5–8 Hz from the experimental set value did not cause a substantial variation in the integrated peak volumes [52,53]. In particular, it was calculated the ratio of the signals at *δ*_H/C_ 5.31/98.5 and 5.77/97.7 ppm, corresponding to 6-*O*- and 4-*O*-α-linked CH-1 Fuc atoms respectively (Figures S12–S14), as previously assigned by 2D-NMR spectroscopy [29]. The obtained data reveal a decrease in 6-*O* *vs.* 4-*O*-regioselectivity with the increase of the glycosylation temperature from −30 °C to 25 °C, even if in all cases the relative amount of 6-*O*-linked α-Fuc branches was much higher with respect to the 4-*O*-linked ones (see Table 1). Concerning Fuc branching stereochemistry, the α/β ratio could not be determined directly by integration of the α- and β-Fuc anomeric signals, because the latter could be not detected unambiguously even in 2D-NMR spectra due to low intensity and/or overlap with the signals of α-Fuc or GlcA and GalNAc units [54]. Nonetheless, an indirect estimation of the α/β ratio was possible by evaluating the β-Fuc branches amount as the difference between the CH_3_-6 and the sum of 6-*O* and 4-*O*-linked α-Fuc CH-1 signal integrations—as the former counts for both α- and β-linked units—according to Equation (2).

(2) $$\alpha /\beta =\frac{\sum I\;\left(CH-1\;\alpha -Fuc\right)}{\frac{I\;\left(CH3-6\;Fuc\right)}{3}-\sum I\;\left(CH-1\;\alpha -Fuc\right)}$$

The obtained data revealed a clear increase of α- over β- stereochemistry with the increase of glycosylation temperature (see Table 1). In particular, α-linked Fuc branches were almost exclusively found on **fCS-ii** and **fCS-iii**, as expected from the α-stereodirecting effect of DMF in fucosylations [46], whereas β-stereochemistry surprisingly prevailed on polysaccharide **fCS-i** obtained with a glycosylation at −30 °C. Evaluation of the weight-averaged molecular mass (*M_w_*) and polydispersity for **fCS-i**–**iii** was performed by HP-SEC-TDA. They all showed a *M_w_* value that was rather lower (7.8–9.2 kDa) with respect to both starting *E. coli* sourced chondroitin (38 kDa) [55] and natural fCSs (55–65 kDa) [56]. This result was expected [55] due to the acid-mediated reactions (methyl esterification, benzylidene ring installation and cleavage as well as glycosylation) employed in the semi-synthetic strategy to **fCS-i**–**iii**.

**Figure 4:**
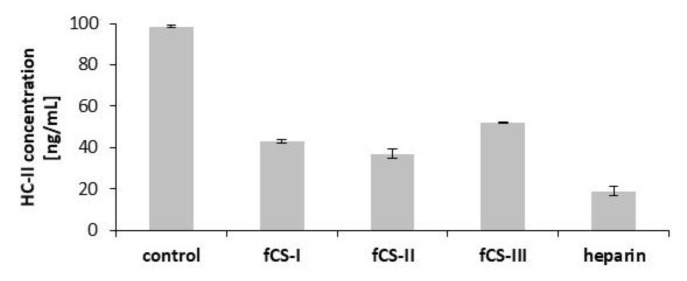
HC-II-mediated anti-factor IIa activity of **fCS-i**–**iii**.

Finally, a very preliminary assay of the anticoagulant activity of the semi-synthetic fCSs polysaccharides was made by evaluating their HC-II-mediated anti-factor IIa activity with respect to heparin representing a positive control. **fCS-i**–**iii** all showed a significant anticoagulant effect (*p* < 0.01), even if with an efficacy from 1.9- to 2.7-fold lower with respect to commercial unfractionated heparin (Figure 4). Such behavior is similar to most of the fCSs species from natural sources assayed up to now and with a *M_w_* close to **fCS-i**–**iii** [56,57,58]. Furthermore, it is worth noting that a recently reported semi-synthetic fCS [29], showing the same sulfation pattern of **fCS-i**–**iii** but with an approximately double amount of Fuc branches attached on the polysaccharide (DF = 0.45) even if with a much lower degree of GalNAc *O*-6 *vs.* *O*-4 regioselectivity, displayed a HC-II-mediated anti-factor IIa activity even slightly higher than heparin. This last result seems to contradict a very recent report on the effect of DF modulation on natural fCS activity, which negatively correlates the number of sulfated fucosyl branches with the HC-II-dependent thrombin inhibitory activation [58]. However, a complete panel of anti-coagulant and anti-thrombotic assays would be much more indicative of the structure–activity relationships of the semi-synthetic fCSs obtained up to now. This work is currently underway and will be published elsewhere in a near future. Nonetheless, the preliminary results here reported already drive the future semi-synthetic work towards the obtainment of further fCS polysaccharides with a high control of not only the sulfation and fucosylation pattern but also of the degree of fucosylation.

## 3. Materials and Methods

### 3.1. General Methods

Commercial grade reagents and solvents were used without further purification, except where differently indicated. The term “deionized water” refers to water purified by a Millipore Milli-Q gradient system. Centrifugations were performed at 4 °C (3500 g, 5 min) with an Eppendorf Centrifuge 5804 R instrument. Measurements of pHs were performed with a Denver Instrument BASIC pH Meter. Dialyses were conducted at 4 °C on Spectra/Por 3.5 kDa cut-off membranes. Freeze-dryings were performed with a 5Pascal Lio 5P 4K freeze dryer. NMR spectra were recorded on a Bruker Avance III HD 400 MHz (^1^H NMR: 400 MHz, ^13^C NMR: 100 MHz) or on a Bruker DRX-600 (^1^H: 600 MHz, ^13^C: 150 MHz) instrument equipped with a cryo probe, in D_2_O (acetone as internal standard, ^1^H: (CH_3_)_2_CO at *δ* 2.22 ppm; ^13^C: (CH_3_)_2_CO at *δ* 31.5 ppm) or DMSO-d_6_ (^1^H: CHD_2_SOCD_3_ at *δ* 2.49 ppm; ^13^C: CD_3_SOCD_3_ at *δ* 39.5 ppm). Standard Bruker software was used for all the experiments. HSQC-DEPT experiments were measured in the ^1^H-detected mode via single quantum coherence with proton decoupling in the ^13^C domain, using data sets of 2048 × 256 points and typically 60 increments. A Viscotek instrument (Malvern) was used to determine molecular mass data.

### 3.2. Preparation of the Products

#### 3.2.1. Example of Glycosylation Reaction

A mixture of polysaccharide acceptor **5** (45.0 mg, 94.3 μmol) and fucosyl donor **10** (226 mg, 0.472 mmol) (or 287 mg, 0.472 mmol **11**) was co-evaporated three times with dry toluene (3 mL each). AW-300 4Å molecular sieves and then DMF (3.4 mL) and CH_2_Cl_2_ (5.6 mL), which were freshly dried over acid-washed 4Å molecular sieves (AW-300 4Å-MS), were added to the mixture under an argon atmosphere. In the case of the reactions on donor **10**, the mixture was stirred at −30 °C (or −10 °C or 25 °C) for 10 min, and then treated with NIS (117 mg, 0.519 mmol) and a 0.50 M solution of TMSOTf in freshly dried CH_2_Cl_2_ (255 μL, 0.127 mmol). In the case of the reactions on donor **11**, only a 0.50 M solution of TMSOTf in freshly dried CH_2_Cl_2_ (24 μL, 11.9 μmol) was added. After 4 h of stirring at −30 °C (or −10 °C or 25 °C), a few drops of triethylamine were added to quench the reaction and then molecular sieves were removed by decantation. By addition of diisopropyl ether (30 mL) a precipitate was formed. It was collected by centrifugation and then dried under vacuum overnight to afford a mixture containing derivative **12-i** (74.0 mg, 164% mass yield).

#### 3.2.2. Example of Benzyl Ether Oxidative Cleavage

Derivative **12-i** (64.6 mg) was suspended in ethyl acetate (2.1 mL) and then treated with a 0.27 M solution of NaBrO_3_ in deionized water (2.4 mL). A 0.24 M solution of Na_2_S_2_O_4_ in deionized water (2.2 mL) was added portionwise over a period of 10 min. The mixture was vigorously stirred at room temperature (r.t.) overnight under visible-light irradiation. A yellowish solid was collected by centrifugation. The supernatant was diluted with ethyl acetate (30 mL) and water (30 mL) and partitioned in a separatory funnel. The aqueous phase was dialyzed and, after freeze-drying, mixed with the previously obtained precipitate to afford derivative **13-i** (38.2 mg, 59% mass yield) as a yellowish amorphous solid.

#### 3.2.3. Example of Sulfation and Global Deprotection

Polysaccharide **8-i** (39.9 mg, 68.7 μmol repeating unit) was dissolved in DMF (700 μL), which was freshly dried over 4 Å molecular sieves. The solution was treated with a 0.71 M solution of pyridine–sulfur trioxide complex in freshly dried DMF (1.9 mL) and then stirred overnight at 50 °C. A cold, saturated NaCl solution in acetone (14 mL) was added to give a yellowish precipitate that was collected by centrifugation and then suspended in deionized water (5.0 mL). The resulting acid mixture (pH ≈ 2) was stirred for 2 h at 50 °C to give a yellowish solution that was then cooled to r.t. and treated with a 4 M NaOH solution to adjust pH to 12. The solution was stirred at r.t. overnight, and then neutralized by dropwise addition of 1 M HCl. Dialysis and subsequent freeze-drying yielded polysaccharide **CS-i** (32.5 mg, 81% mass yield) as a white amorphous solid.

In the case of reactions on **13-i**–**iv**, the yellowish precipitate obtained by centrifugation was suspended in deionized water (4.3 mL) and treated with 2:1 *v*/*v* aqueous 1 M LiOH–30% H_2_O_2_ (2.1 mL, pH ≈ 10). The resulting solution was stirred at r.t. overnight, then neutralized with 1 M HCl, and finally treated with 1 M NaCl (4.2 mL). After 1 h stirring at r.t., the solution was dialyzed, freeze-dried and the resulting amorphous solid was re-dissolved in deionized water (1.5 mL) and further purified by filtration through a Sep-pak C-18 cartridge (elution with deionized water) to give, after freeze-drying, **fCS-i**–**iv** as white waxy solids.

#### 3.2.4. Study of Chondroitin Polysaccharide Stability under Different Hydrolysis Reaction Conditions

Chondroitin sodium salt from *Escherichia coli* O5:K4:H4 [28] (1.259 g, 3.140 mmol repeating unit) was dissolved in deionized water (60 mL) and then treated with AcOH (3.6 mL). The obtained solution (pH 4.6) was stirred at 100 °C. At different times, 15 mL aliquots were collected, cooled to -28 °C and freeze-dried. The obtained solid residues were dissolved in deionized water (30 mL) and dialyzed. After freeze-drying, the samples were re-dissolved in water (15 mL) and eluted through a Dowex 50 WX8 (H^+^ form) column. The eluates were freeze-dried to give solid samples that were then weighted and their molecular mass and polydispersity data measured by Viscotek analysis.

Alternatively, two samples of chondroitin sodium salt (1.083 g, 2.701 mmol repeating unit) were dissolved in deionized water (47 mL) and then treated with some drops of 4 M HCl. The obtained solution (pH 1.1 or pH 1.6) was stirred at 70 °C or 50 °C, respectively. At different times, 15 mL aliquots were collected, neutralized with 1 M NaOH and dialyzed. After freeze-drying, the samples were re-dissolved in water (15 mL) and eluted through a Dowex 50 WX8 (H^+^ form) column. The eluates were freeze-dried to give solid samples that were then weighted and their molecular mass and polydispersity data measured by HP-SEC-TDA analysis.

Alternatively, chondroitin sodium salt was transformed into its free acid form by treatment with Dowex 50 WX8 (H^+^ form) as already described. [30] The acid polysaccharide (1.837 g, 4.847 mmol repeating unit) was dissolved in deionized water (125 mL) to give a solution (pH 2.1) that was stirred at r.t. At different times, 30 mL aliquots were collected, neutralized with 1 M NaOH and dialyzed. After freeze-drying, the samples were re-dissolved in water (30 mL) and eluted through a Dowex 50 WX8 (H^+^ form) column. The eluates were freeze-dried to give solid samples that were then weighted and their molecular mass and polydispersity data measured by HP-SEC-TDA analysis.

#### 3.2.5. Study of Benzylidene Acetal vs. Glycosidic Bond Cleavage on Chondroitin Derivative **2**

Chondroitin derivative **2** [29] (101 mg, 179 μmol repeating unit) was dissolved in 9:1 *v*/*v* AcOH-H_2_O (3.0 mL) and the obtained solution was stirred at 50 °C. At different times, 500 μL aliquots were collected, cooled to r.t., diluted with water (10 mL) and dialyzed. After freeze-drying, the obtained solid residues were weighted and subjected to ^1^H NMR analysis in DMSO-d_6_.

Alternatively, derivative **2** [29] (92.7 mg, 164 μmol repeating unit) was dissolved in CH_3_CN (1.8 mL) and then treated with 1,4-dithiothreitol (DTT, 127 mg, 820 μmol) and (+)-camphor-10-sulfonic acid (CSA, 47.6 mg, 205 μmol). The solution was stirred at r.t. At different times, 400 μL aliquots were collected, cooled to r.t., diluted with water (10 mL) and dialyzed. After freeze-drying, the obtained solid residues were weighted and subjected to ^1^H NMR analysis in DMSO-d_6_.

#### 3.2.6. Polysaccharide Derivative **5**

A solution of **2** [29] (99.6 mg, 176 μmol repeating unit) in 9:1 *v*/*v* AcOH-H_2_O (3.0 mL) was stirred at 50 °C for 48 h. It was then cooled to r.t. and diluted with H_2_O (15 mL). The solution was dialyzed to give, after freeze-drying, derivative **5** (78.1 mg, 78% mass yield) as a white powder.

#### 3.2.7. Polysaccharide Derivative **8-i**

Chondroitin derivative **2** [29] (40.1 mg, 71.0 μmol repeating unit) was co-evaporated three times with dry toluene (4 mL each). The residue was further dried under vacuum for 2 h and then mixed with AW-300 4Å-MS under an argon atmosphere. The mixture was treated with 1:1 *v*/*v* CH_2_Cl_2_–CH_3_CN (4.2 mL), that was freshly dried over AW-300 4Å-MS. The polysaccharide was completely dissolved in few minutes. The mixture was cooled to 0 °C and then treated with Et_3_SiH (136 μL, 0.88 mmol) and BF_3_⸱OEt_2_ (18.0 μL, 142 μmol). The mixture was stirred at r.t. for 4 h, then the reaction was worked up by neutralization with triethylamine and then treated with ethyl acetate (15 mL). The obtained precipitate was collected by centrifugation and then dried under vacuum overnight to afford **8-i** (19.2 mg, 48% mass yield) as a yellowish powder.

#### 3.2.8. Polysaccharide Derivative **8-ii**

Chondroitin derivative **2** [29] (44.3 mg, 78.3 μmol repeating unit) was co-evaporated three times with dry toluene (4 mL each). The residue was further dried under vacuum for 2 h and then mixed with AW-300 4Å-MS under an argon atmosphere. The mixture was treated with THF (2.0 mL), that was freshly dried over AW-300 4Å-MS. The polysaccharide was finely suspended after four hours stirring at 50 °C. The mixture was then cooled to r.t. and treated with a 1.0 M solution of BH_3_·THF complex in dry THF (783 μL, 783 μmol) and a 0.55 M solution of TMSOTf in dry THF (46 μL, 25.5 μmol). The mixture was stirred at r.t. overnight, then the reaction was worked up by neutralization with triethylamine and then addition of methanol (3 mL). The solution obtained by mechanical separation from the molecular sieves was concentrated by roto-evaporation. The residue was dissolved in DMSO (1 mL) and then treated with acetone (4 mL). The obtained precipitate was collected by centrifugation and then dried under vacuum overnight to afford **8-ii** (22.0 mg, 50% mass yield) as a brownish amorphous solid.

#### 3.2.9. **fCS-i**–**iii**

**fCS-i**–**iii** were obtained from polysaccharide acceptor **5** and fucosyl donor **10** through a sequence of reactions (glycosylation, benzyl ether oxidative cleavage, sulfation and global deprotection) performed as indicated in paragraphs 3.2.1–3.2.3. In particular, 22.6 mg **fCS-i** were obtained starting from 45.0 mg **5** and 226 mg **10**, 19.2 mg **fCS-ii** were obtained from 36.8 mg **5** and 185 mg **10**, and 16.3 mg **fCS-iii** were obtained from 28.1 mg **5** and 141 mg **10**.

### 3.3. Determination of Molecular Mass Data

Molecular mass analyses were performed by a Viscotek high-performance size-exclusion chromatographic (HP-SEC) system equipped with an integrated gel permeation chromatography system (GPCmax VE 2001,Viscotek, Malvern, Egham, UK) and a triple detector array module (TDA 305, Viscotek, Malvern, Egham, UK) including a refractive index detector (RI), a four-bridge viscosimeter (VIS), and a laser detector (LS) made of a right-angle light scattering (RALS) detector and a low-angle light scattering (LALS) one. The employed analytical method was already extensively described [39].

### 3.4. Anti-Coagulant Activity

Anti HC-IIa assay was performed using a sandwich enzyme immunoassay (USCN, Life Science, Wuhan, China). The microtiter plate provided in this ELISA kit had been pre-coated with an antibody specific to HC-II. Standards or samples were then added to the appropriate microtiter plate wells with a biotin-conjugated antibody specific to HC-II. Next, avidin conjugated to Horseradish Peroxidase (HRP) was added to each microplate well and incubated. Successively, TMB substrate solution was added, only those wells that contain HC-II, biotin-conjugated antibody and enzyme-conjugated avidin exhibited a change in color. The enzyme-substrate reaction was terminated by the addition of sulfuric acid solution and the color change was measured spectrophotometrically at a wavelength of 450 ± 10 nm. The concentration of HCII in the samples was then determined by comparing the optical density (OD) of the samples to the standard curve.

## 4. Conclusions

A semi-synthetic access to fCS polysaccharides carrying sulfated Fuc branches at position *O*-6 of GalNAc units has been reported here. It employs *E. coli* O5:K4:H4 sourced chondroitin polysaccharide as starting material on which different sequences of chemical steps have been attempted in order to insert sulfated Fuc branches regioselectively at a single site of GalNAc units. The successful strategy relied upon two key steps that were carefully optimized: (i) a chemoselective cleavage of benzylidene acetal moieties protecting GalNAc *C*-4,6-positions to restore the diol without a significant breakage of glycosidic bonds, and (ii) regioselective glycosylation of *O*-6 site of the restored diol with a Fuc thioglycoside donor. The semi-synthetic fCS polysaccharides obtained after further steps were subjected to structural characterization by NMR and HP-SEC-TDA and to a preliminary *in vitro* anti-coagulant test.

## Figures and Tables

**Table 1:** Yield and structural data of **fCS-i**–**iv**.

Sample	Fucosyl Donor, Glycosylation Temperature	Yield ^1^ [%]	DF ^2^	GalNAc *O*-6/*O*-4 Branching Site Ratio ^3^	α/β Fuc Ratio ^3^	*M_w_*^4^ [kDa]	*M_w_*^4^/*M_n_*^5^
**fCS-i**	**10**, −30 °C	50	0.21	only 6-*O*-linked	1:3	9.2	1.31
**fCS-ii**	**10**, −10 °C	52	0.20	9:1	9:1	9.1	1.31
**fCS-iii**	**10**, 25 °C	58	0.29	4:1	19:1	7.8	1.19
**fCS-iv**	**11**, −10 °C	51	0.04	n.d. ^6^	n.d. ^6^	n.d. ^6^	n.d. ^6^

^1^ Overall mass yield determined with respect to starting glycosyl acceptor **5**. ^2^ Determined by ^1^H NMR integration of Fuc methyl and GalNAc acetyl signals. ^3^ Estimated by DEPT-HSQC integration (see text for details). ^4^ *M_w_* = weight-averaged molecular mass. ^5^ *M_n_* = number-averaged molecular mass. ^6^ Not determined.

## Supplementary Materials

The following are available online at https://www.mdpi.com/1660-3397/17/12/655/s1, Figure S1: ^1^H NMR spectrum of **8-i**, Figure S2: ^1^H NMR spectrum of **8-ii**, Figure S3: ^1^H and DEPT-HSQC NMR spectra of **CS-i**, Figure S4: ^1^H and DEPT-HSQC NMR spectra of **CS-ii**, Figure S5: Stacked ^1^H NMR spectra of aliquots taken at different times from hydrolysis reaction **2**→**5** with 90% aq. AcOH, Figure S6: Stacked ^1^H NMR spectra of aliquots taken at different times from hydrolysis reaction **2**→**5** with DTT and CSA, Figure S7: ^1^H NMR spectrum of **12-i**, Figure S8: ^1^H NMR spectrum of **12-ii**, Figure S9: ^1^H NMR spectrum of **12-iii**, Figure S10: ^1^H NMR spectrum of **12-iv**, Figure S11: Stacked ^1^H NMR spectra of **fCS-i**–**iv**, Figure S12: DEPT-HSQC NMR spectrum of **fCS-i**, Figure S13: DEPT-HSQC NMR spectrum of **fCS-ii**, Figure S14: DEPT-HSQC NMR spectrum of **fCS-iii**.
