# Supplementary material for: A Study for the Access to a Semi-synthetic Regioisomer of Natural Fucosylated Chondroitin Sulfate with Fucosyl Branches on N-acetyl-Galactosamine Units
Source: Mar Drugs. 2019 Nov 21;17(12):655. doi: 10.3390/md17120655 (PMC6950142; doi:10.3390/md17120655)
Supplement: Supplementary file 1 [file marinedrugs-17-00655-s001.pdf]

Supporting Information

A STUDY FOR THE ACCESS TO A SEMI-SYNTHETIC REGIOISOMER OF NATURAL FUCOSYLATED CHONDROITIN SULFATE WITH FUCOSYL BRANCHES ON *N*-ACETYL-GALACTOSAMINE UNITS

GIULIA VESSELLA,[1] SERENA TRABONI,[1] ANNA V.A. PIROZZI,[2] ANTONIO LAEZZA,[3] ALFONSO IADONISI,[1] CHIARA SCHIRALDI,[2] EMILIANO BEDINI,[1]

[1]*Department of Chemical Sciences, University of Naples Federico II, Complesso Universitario Monte S.Angelo, via Cintia 4, I-80126 Napoli, Italy*

[2] *Department of Experimental Medicine, Section of Biotechnology, University of Campania "Luigi Vanvitelli", via de Crecchio 7, I-80138 Napoli, Italy*

[3] *Department of Sciences, University of Basilicata, viale dell'Ateneo Lucano 10, I-85100 Potenza, Italy*

Table of Contents

Figure S1: ^{1}H-NMR spectrum of **8-i** S-3

Figure S2: ^{1}H-NMR spectrum of **8-ii** S-3

Figure S3: ^{1}H and DEPT-HSQC NMR spectra of **CS-i** S-4

Figure S4: ^{1}H and DEPT-HSQC NMR spectra of **CS-ii** S-5

Figure S5: Stacked ^{1}H-NMR spectra of aliquots taken at different times from hydrolysis reaction **2**→**5** with 90% aq. AcOH S-6

Figure S6: Stacked ^{1}H-NMR spectra of aliquots taken at different times from hydrolysis reaction **2**→**5** with DTT and CSA S-6

Figure S7: ^{1}H-NMR spectrum of **12-i** S-7

Figure S8: ^{1}H-NMR spectrum of **12-ii** S-7

Figure S9: ^{1}H-NMR spectrum of **12-iii** S-8

Figure S10: ^{1}H-NMR spectrum of **12-iv** S-8

Figure S11: Stacked ^{1}H-NMR spectra of **fCS-i-iv** S-9

Figure S12: DEPT-HSQC NMR spectrum of **fCS-i** S-10

Figure S13: DEPT-HSQC NMR spectrum of **fCS-ii** S-11

Figure S14: DEPT-HSQC NMR spectrum of **fCS-iii** S-12

References S-12

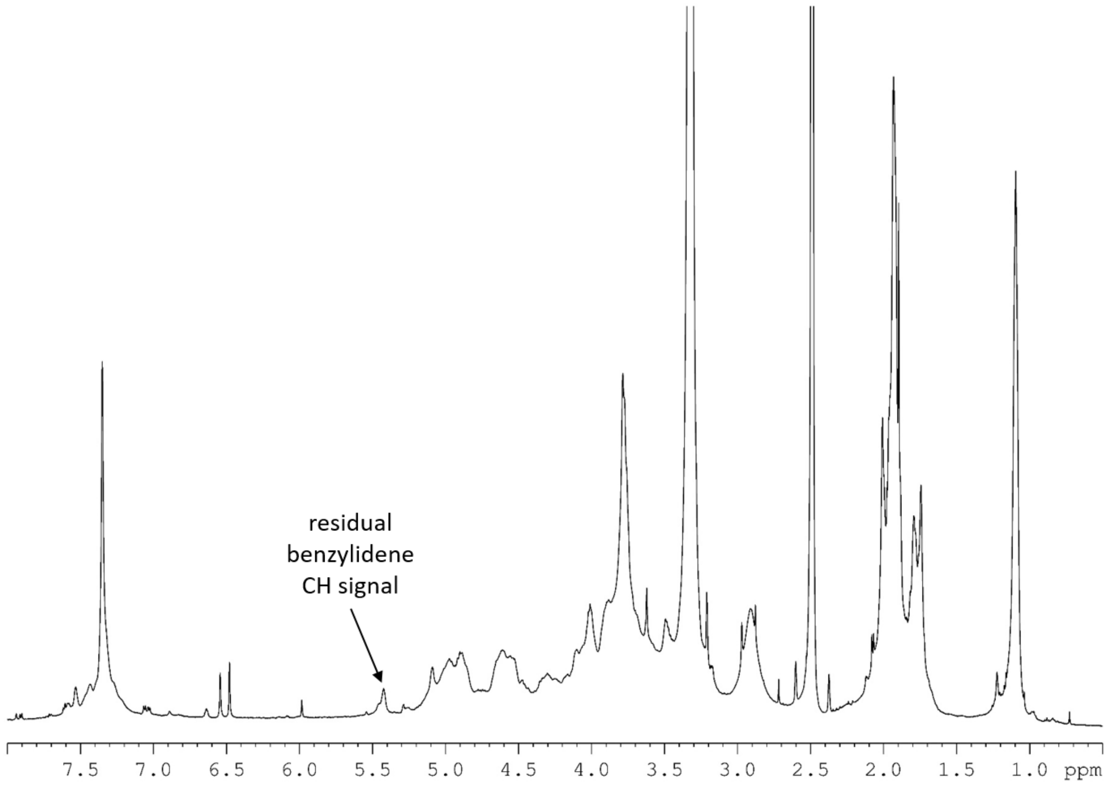

Figure S1: ^{1}H-NMR spectrum (600 MHz, DMSO-d_6, 298 K) of **8-i**

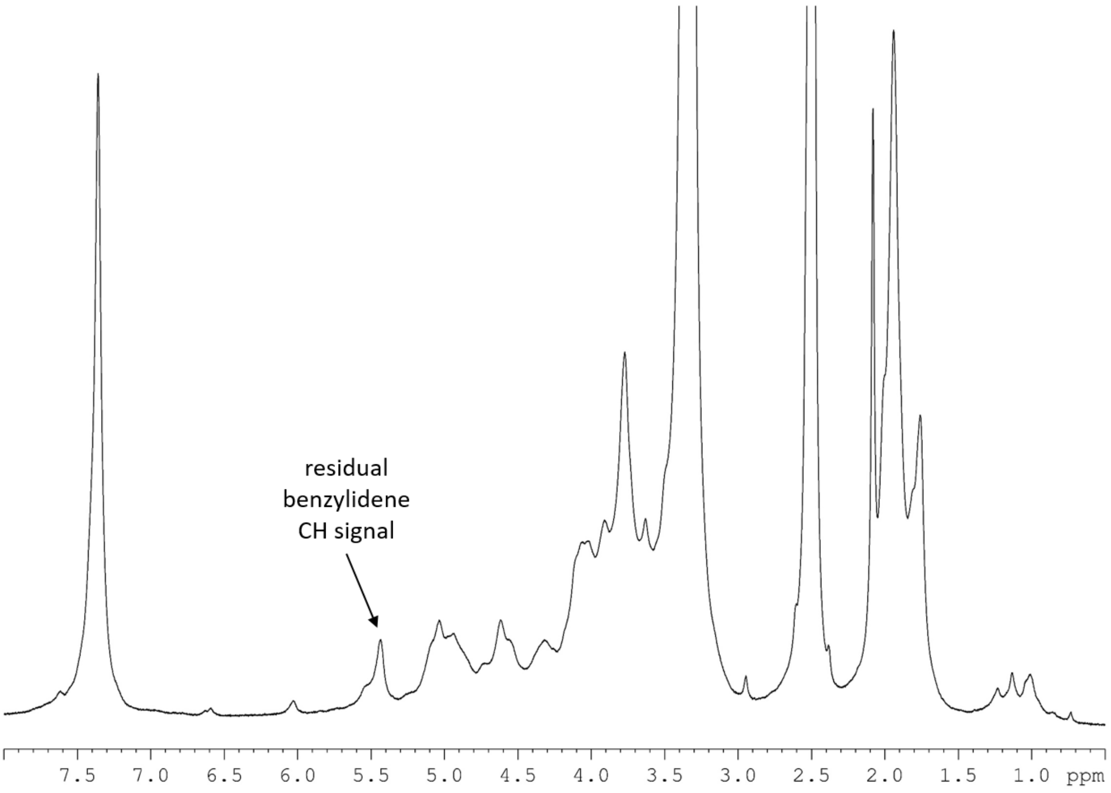

Figure S2: ^{1}H-NMR spectrum (600 MHz, DMSO-d_6, 298 K) of **8-ii**

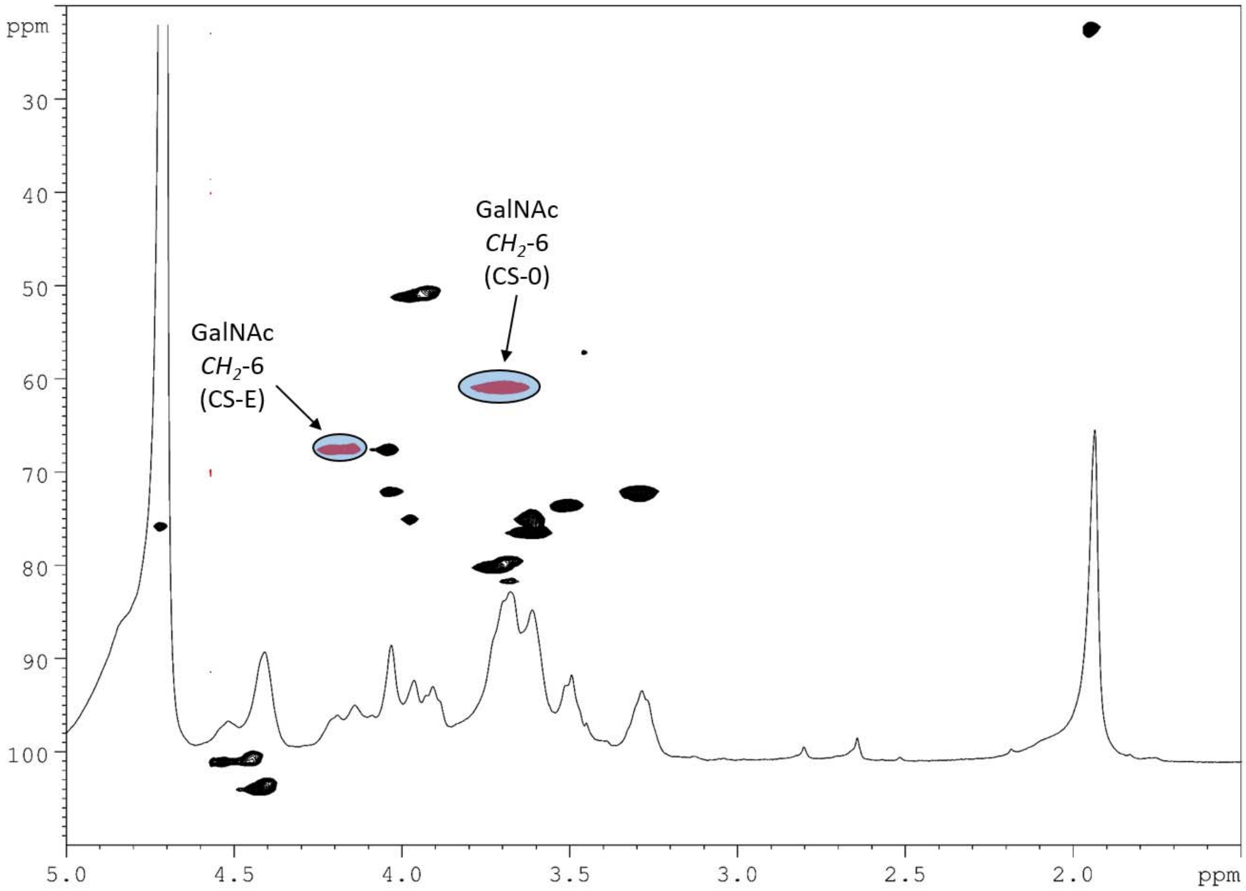

Figure S3: ^{1}H and DEPT-HSQC NMR spectra (400 MHz, D_2O, 298K) of **CS-i**
(densities enclosed in the highlighted areas were integrated for CS-0/CS-E ratio estimation)

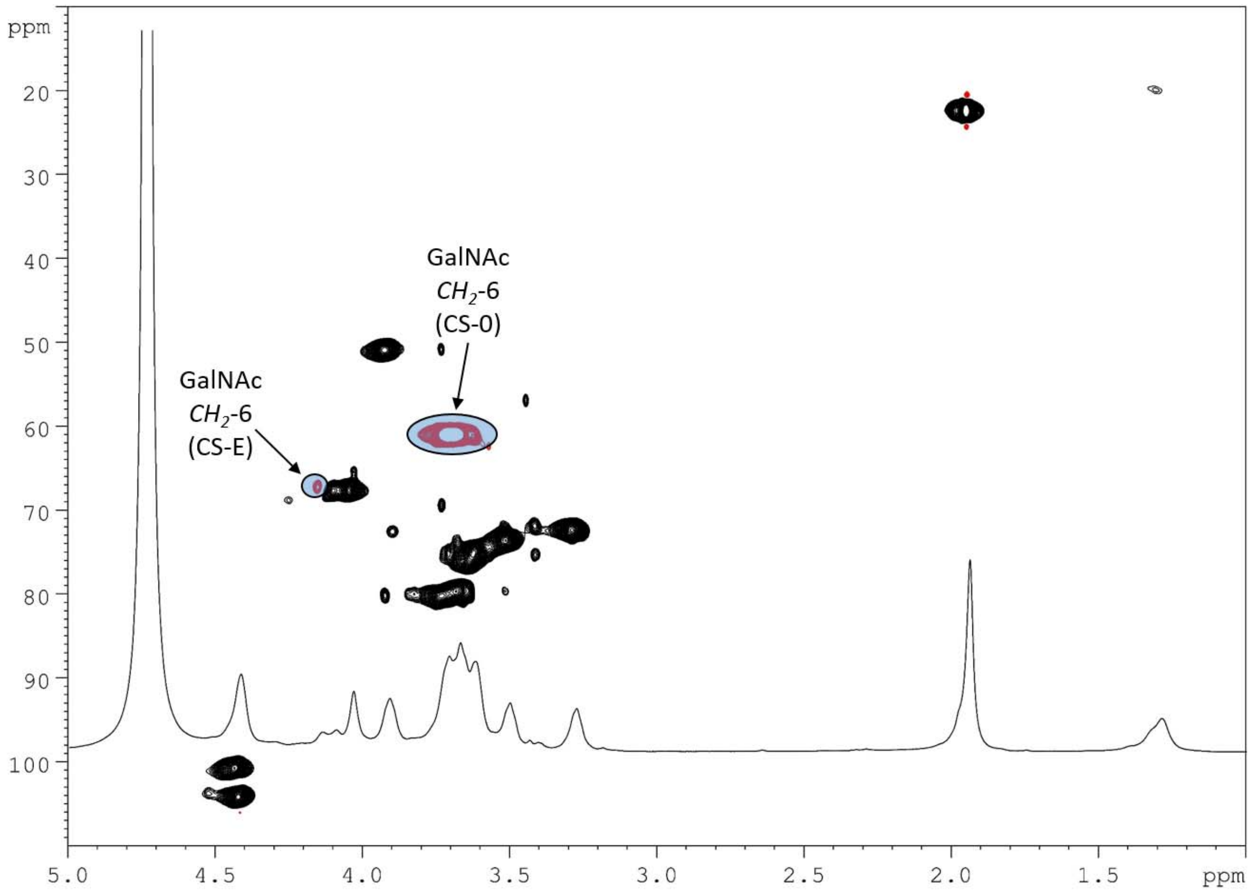

Figure S4: ^{1}H and DEPT-HSQC NMR spectra (400 MHz, D_2O, 298K) of **CS-ii**
(densities enclosed in the highlighted areas were integrated for CS-0/CS-E ratio estimation)

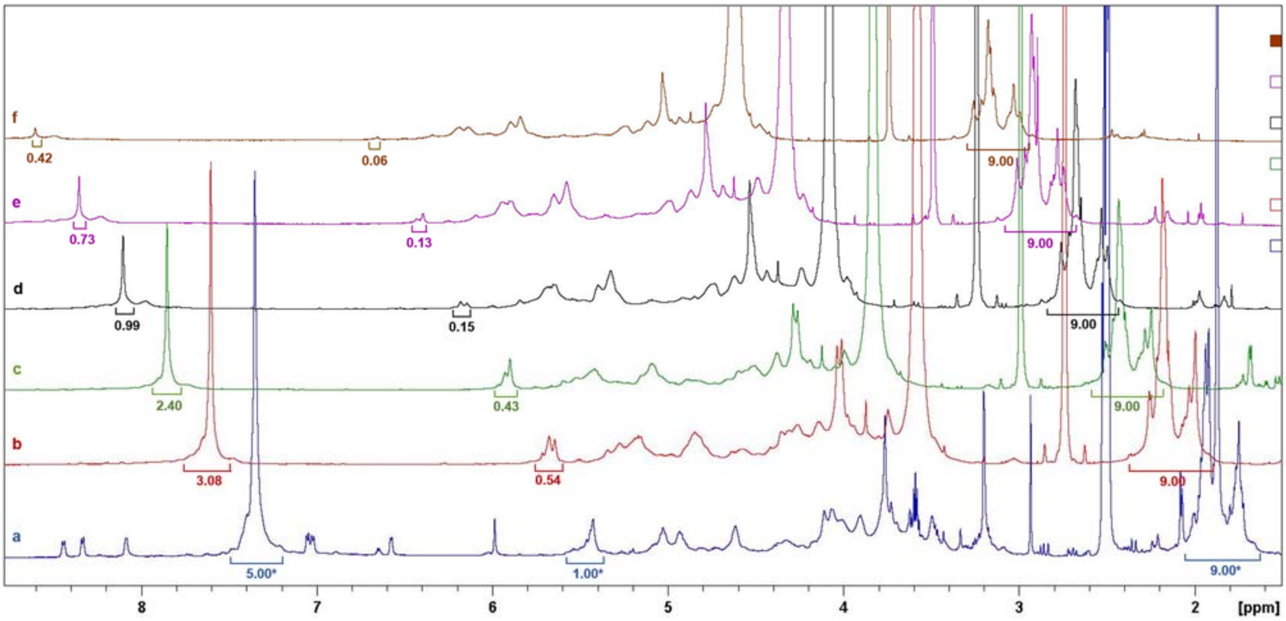

Figure S5: Stacked (with a $\Delta\delta = 0.25$ ppm shift) ^{1}H-NMR spectra (600 MHz, DMSO-d_6, 298 K) of aliquots taken at different times (a: 0, b:3, c:6, d:24, e:42, f:66 hours) from hydrolysis reaction **2→5** with 90% aq. AcOH. Integration values are relative to signals, from left to right, of benzylidene ring protons at δ_H 7.5-7.2, of benzylidene methine proton at 5.45-5.35 ppm, and of *O*- and *N*-acetyl methyl protons at δ_H 2.0-1.7 ppm (integration of *O*- and *N*-acetyl signal for t=0 hours spectrum was taken from literature[S1] due to superimposition with other peaks)

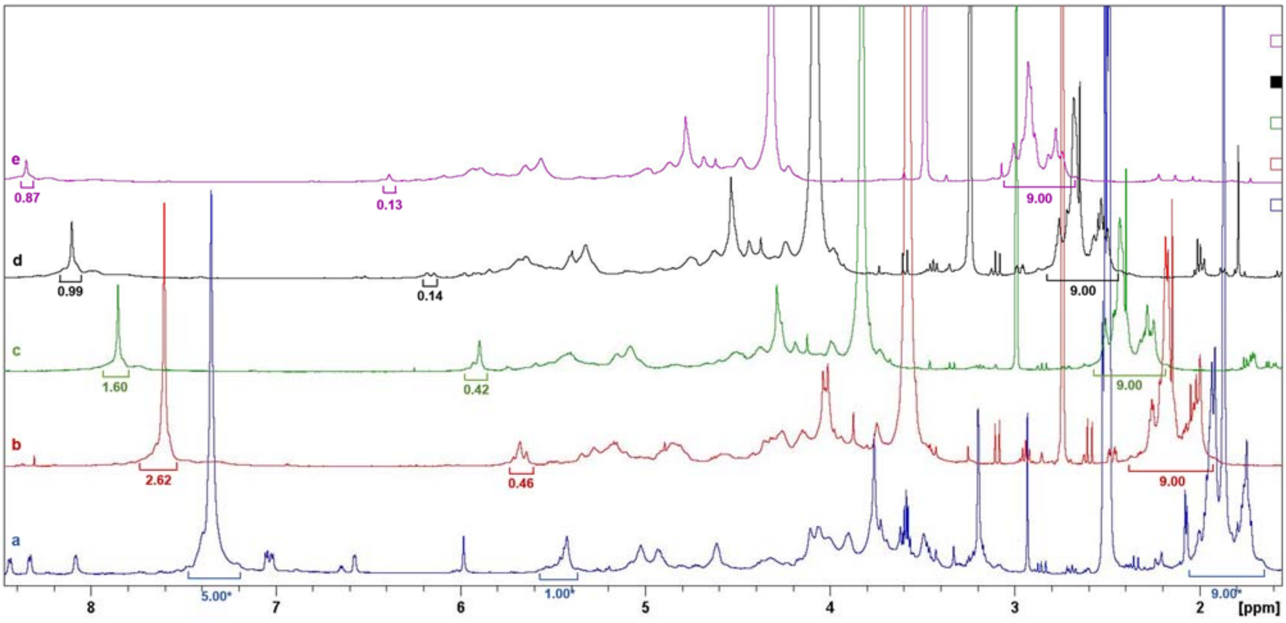

Figure S6: Stacked (with a $\Delta\delta = 0.25$ ppm shift) ^{1}H-NMR spectra (600 MHz, DMSO-d_6, 298 K) of aliquots taken at different times (a: 0, b:5, c:25, d:49, e:73 hours) from hydrolysis reaction **2→5** with DTT and CSA. Integration values are relative to signals, from left to right, of benzylidene ring protons at δ_H 7.5-7.2, of benzylidene methine proton at 5.45-5.35 ppm, and of *O*- and *N*-acetyl methyl protons at δ_H 2.0-1.7 ppm (*integration of signals for t=0 hours spectrum was imposed from literature[S1] due to superimposition with other peaks)

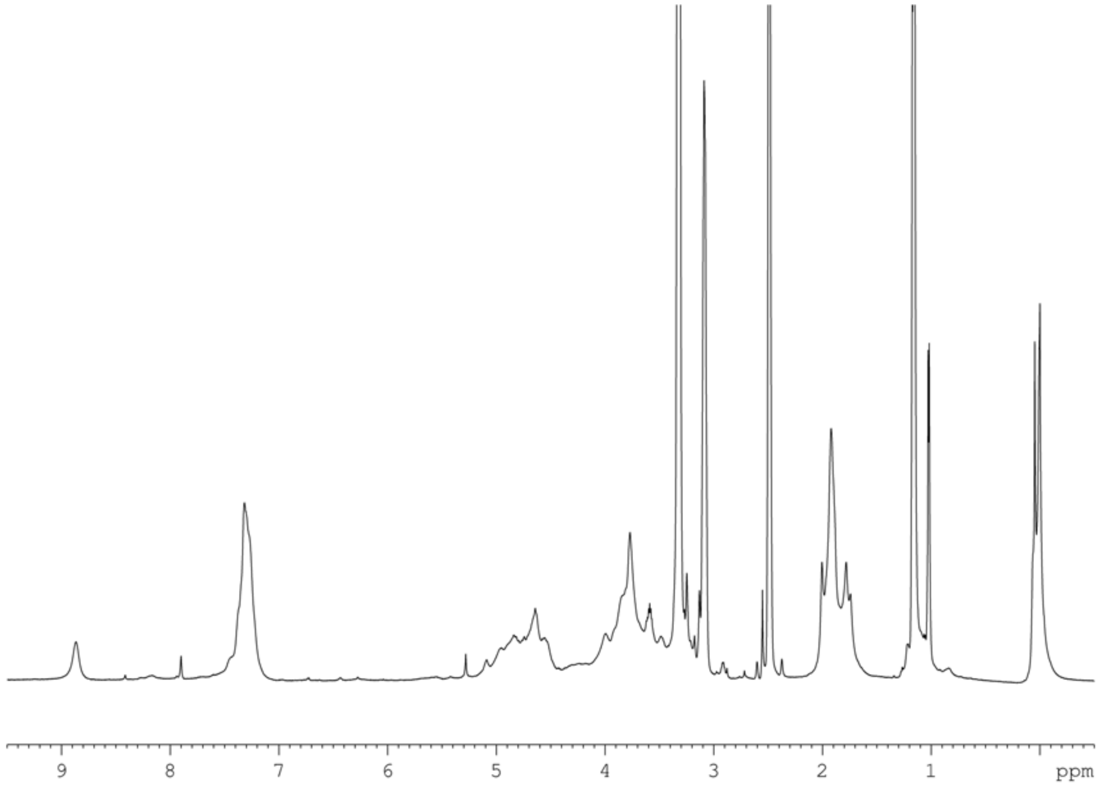

Figure S7: ^{1}H-NMR spectrum (600 MHz, DMSO-d_6, 298 K) of **12-i**

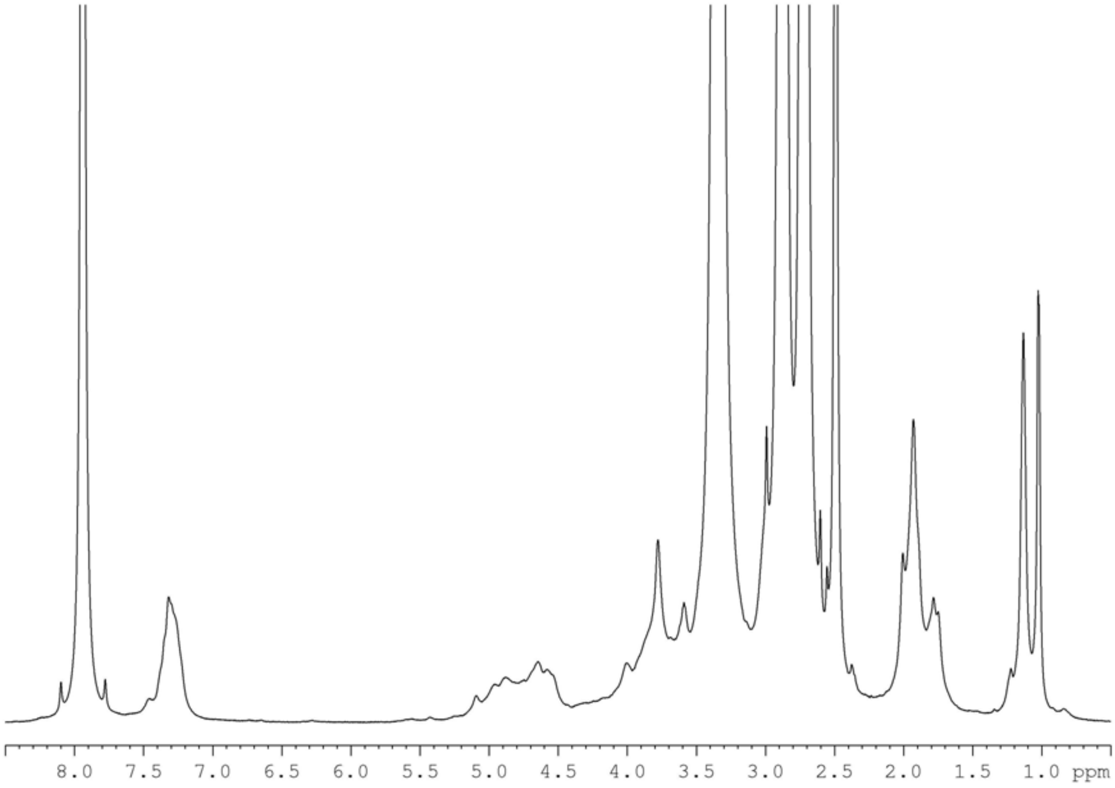

Figure S8: ^{1}H-NMR spectrum (600 MHz, DMSO-d_6, 298 K) of **12-ii**

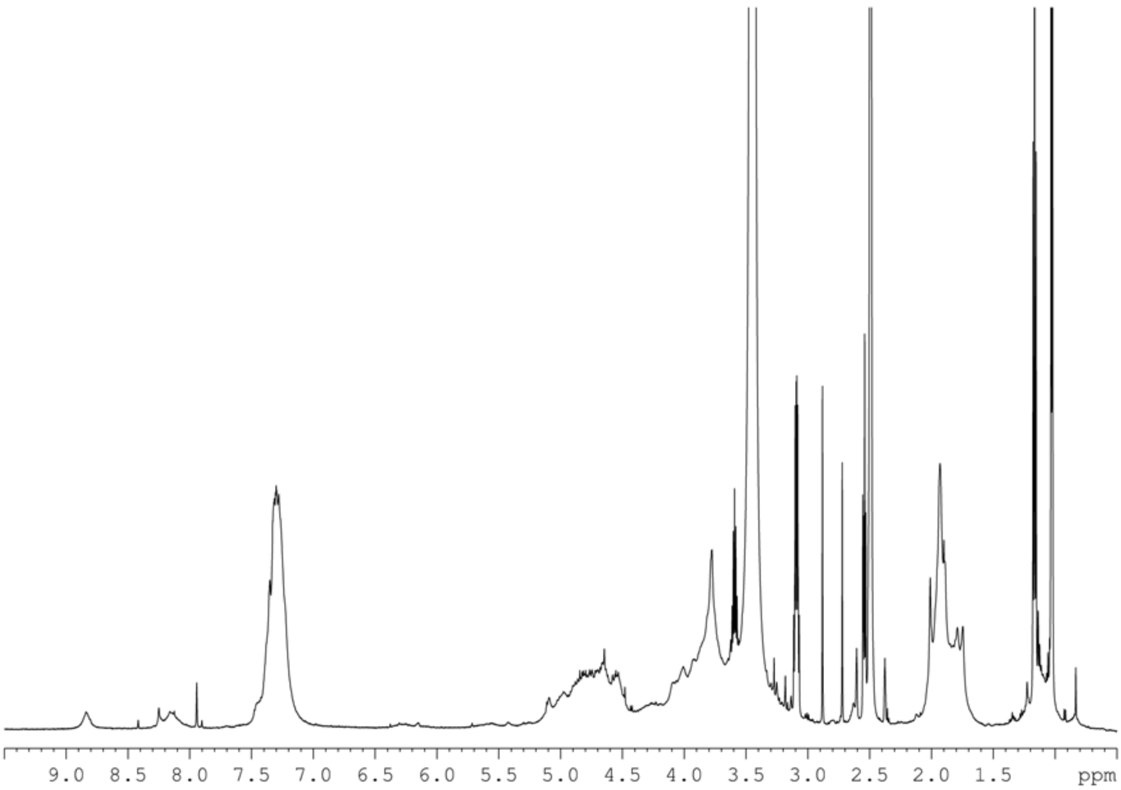

Figure S9: ^{1}H-NMR spectrum (600 MHz, DMSO-d_6, 298 K) of **12-iii**

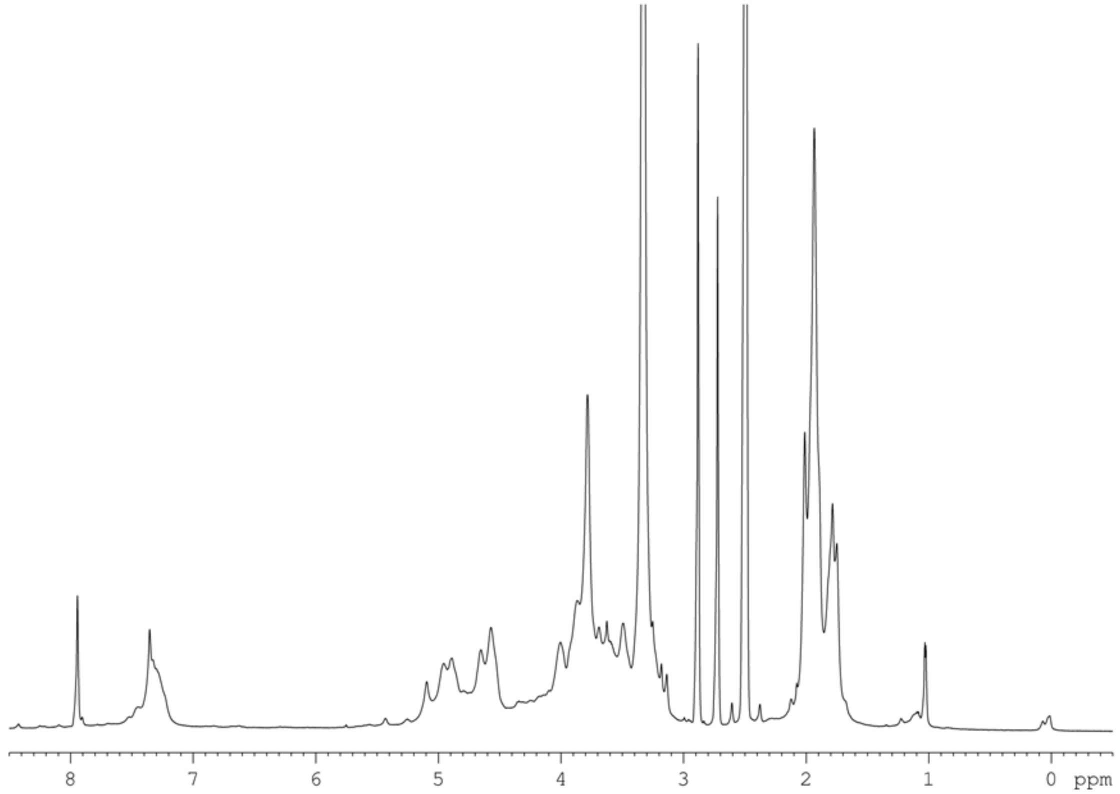

Figure S10: ^{1}H-NMR spectrum (600 MHz, DMSO-d_6, 298 K) of **12-iv**

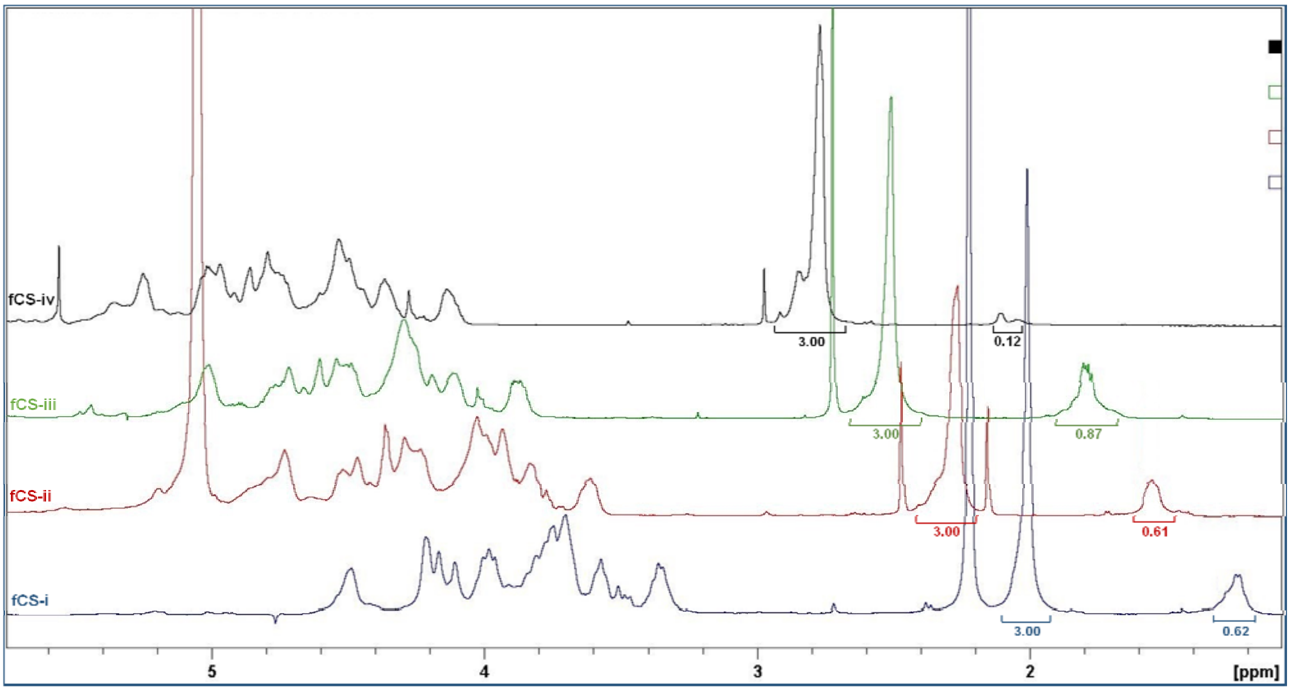

Figure S11: Stacked (with a $\Delta\delta = 0.25$ ppm shift) ^{1}H-NMR spectra (600 MHz, D_2O, 298 K) of **fCS-i-iv**. Integration values are relative to signals, from left to right, of *N*-acetyl methyl protons at δ_H 2.10-1.95 ppm and of Fuc methyl protons at δ_H 1.35-1.20 ppm

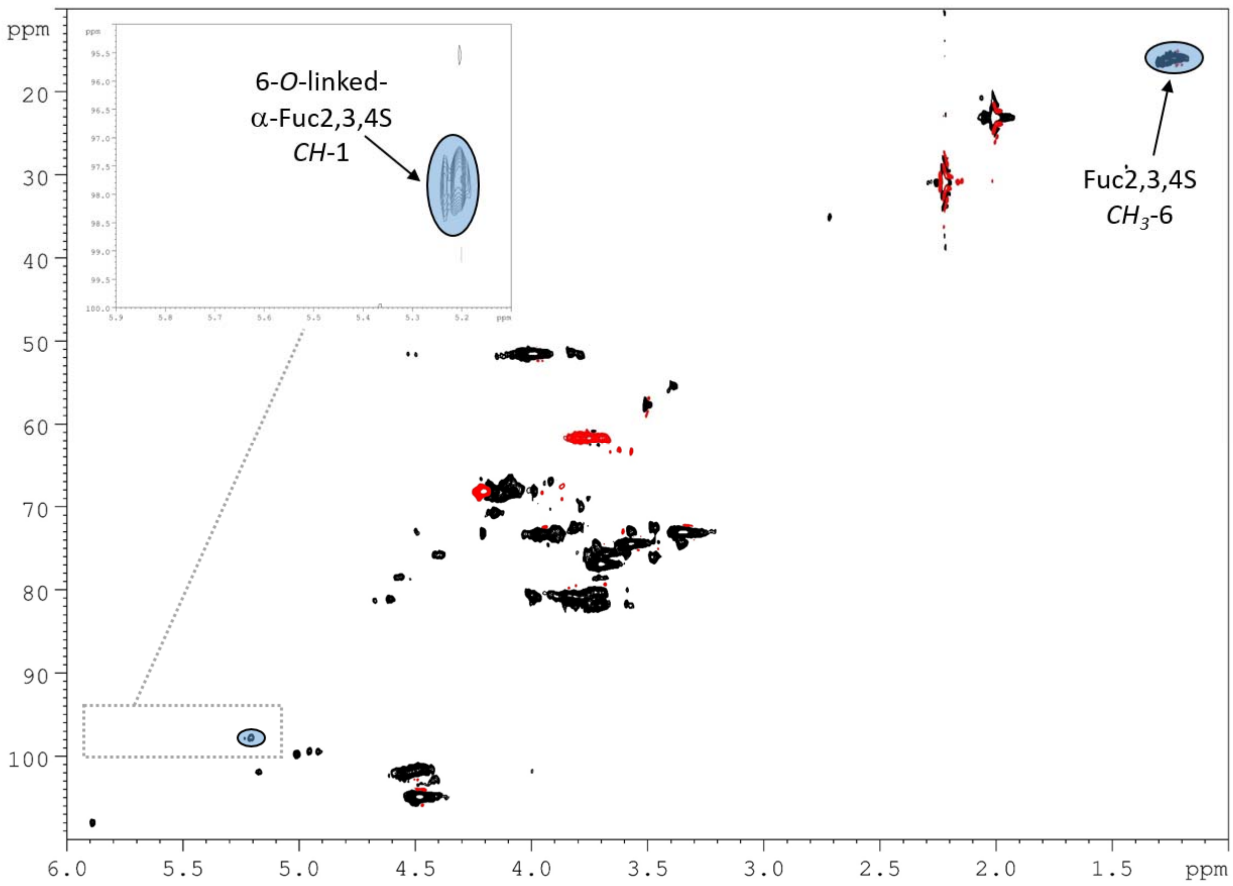

Figure S12: DEPT-HSQC NMR spectrum (600 MHz, D_2O, 298K) of **fCS-i**

(densities enclosed in the highlighted areas were integrated for α/β Fuc and GalNAc *O*-6/*O*-4 branching site ratio)

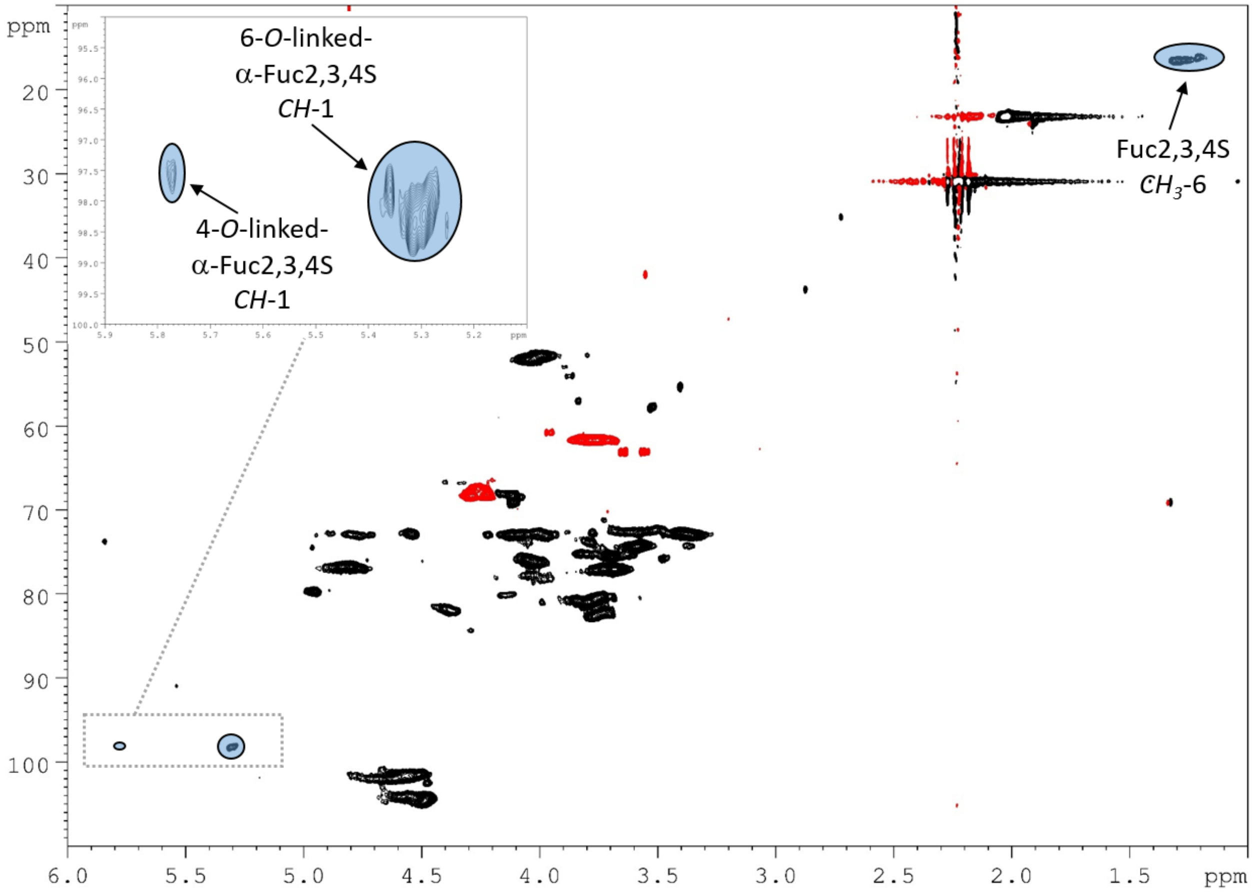

Figure S13: DEPT-HSQC NMR spectrum (600 MHz, D_2O, 298K) of **fCS-ii**

(densities enclosed in the highlighted areas were integrated for α/β Fuc and GalNAc *O*-6/*O*-4 branching site ratio)

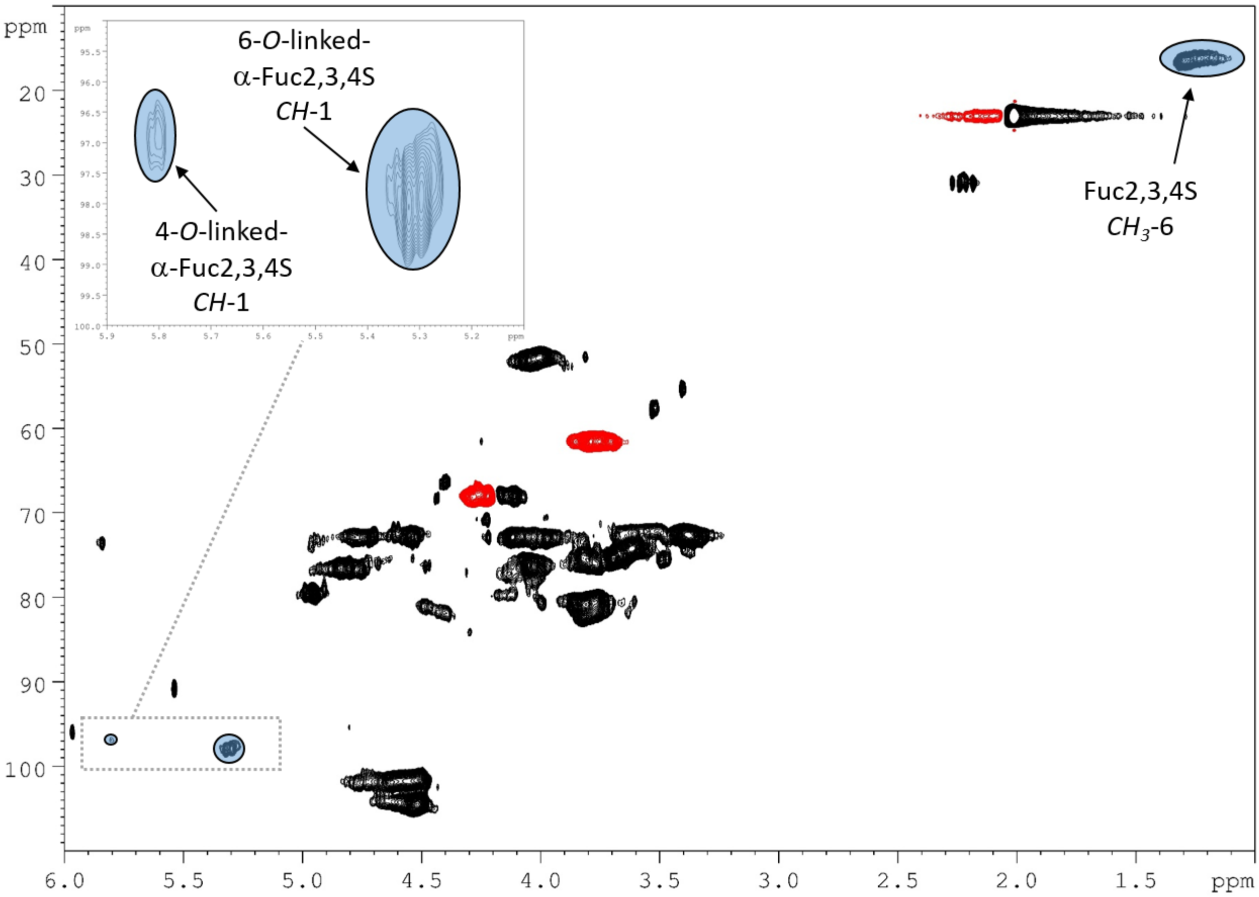

Figure S14: DEPT-HSQC NMR spectrum (600 MHz, D_2O, 298K) of **fCS-iii**
(densities enclosed in the highlighted areas were integrated for α/β Fuc and GalNAc *O*-6/*O*-4 branching site ratio)

References

S1. A. Laezza, A. Iadonisi, A. V. A. Pirozzi, P. Diana, M. De Rosa, C. Schiraldi, M. Parrilli, E. Bedini, *Chem. Eur. J.* **2016**, *22*, 18215–18226
